# Peripheral artery disease recognition, diagnosis, and management in general practice in the Republic of Ireland and England: an online survey

**DOI:** 10.3399/BJGPO.2023.0150

**Published:** 2024-06-26

**Authors:** Judit Konya, Sinead TJ McDonagh, Peter Hayes, Sebastian Debus, Victor Aboyans, Christopher E Clark, for the EuroPAD investigators

**Affiliations:** 1 Exeter Collaboration for Academic Primary Care, Faculty of Health and Life Sciences, University of Exeter Medical School, Smeall Building, Exeter, UK; 2 Discipline of General Practice, School Of Medicine, University of Limerick, Limerick, Ireland, UK; 3 Department for Vascular Medicine (Vascular Surgery; Angiology; Endovascular Therapy), University Heart & Vascular Center Hamburg, Hamburg, Germany; 4 Department of Cardiology, Dupuytren University Hospital, and Inserm U1094 and IRD, Limoges, France

**Keywords:** peripheral arterial disease, blood pressure, cardiovascular diseases, general practitioners, primary healthcare

## Abstract

**Background:**

Peripheral artery disease (PAD) is common and associated with future cardiovascular events. PAD is underdiagnosed, which limits opportunities to address secondary prevention of cardiovascular disease. It is unknown how closely guidelines for detection of PAD are followed in primary care.

**Aim:**

To survey GPs’ attitudes to diagnosis and follow-up of patients with PAD.

**Design & setting:**

Online survey of GPs in England and the Republic of Ireland (RoI).

**Method:**

GPs’ approaches to management of PAD were assessed using likelihood ratings (scales of 0–10) and discrete questions. Findings were summarised as proportions, or median and interquartile ranges (IQR).

**Results:**

In total, 111 responses were analysed; 68 (61%) from England and 43 (39%) from the RoI. Considering a hypothetical patient at risk of PAD, likelihood of GPs enquiring about PAD symptoms (leg pains: 3/10 or erectile dysfunction: 2/10) was low. GPs in the RoI compared with GPs in England more often examined the heart (10/10 versus 7/10) or carotid vessels (5/10 versus 1/10). Lower limb pulses were palpated in response to symptoms or signs of PAD. In England 25% of practitioners, and in the RoI 55% of practitioners, reported that they do not measure ankle-brachial index (ABI).

**Conclusion:**

Currently, detection of PAD is generally triggered by ‘classical’ leg claudication symptoms, while known vascular risk factors appear to elicit little consideration. ABI measurement is not performed by many practitioners, suggesting that a proportion of vascular referrals must be based on history and examination findings alone. Opportunities to recognise PAD are missed.

## How this fits in

Peripheral arterial disease (PAD) is poorly understood by the public, and it is not known how closely appropriate guidelines to diagnose and manage PAD are followed in general practice. Our online survey of GPs in England and the RoI aims to fill this knowledge gap and highlights the opportunity for a better detection rate of the condition.

## Introduction

Peripheral artery disease (PAD) affects up to one-quarter of adults aged >80 years. Overall, global prevalence is 6% for people aged >65 years and is rising.^1,2^ It is a serious condition, leading to critical limb ischaemia, limb loss, or death, thus presenting a substantial healthcare burden. Furthermore, PAD is also a strong indicator of future major adverse cardiovascular events.^3,4^ Both symptomatic and asymptomatic PAD are associated with impaired quality of life and physical function.^5,6^ Leg symptoms vary and are not confined to those classically recognised as intermittent claudication. Therefore, a high index of suspicion is required to consider diagnoses of PAD.^7^ The condition is underdiagnosed, perhaps owing to the heterogeneity of presenting symptoms,^8^ despite the existence of national and international guidelines for diagnosis and management of PAD.^9,10^

Diagnosis of PAD is important for both short and long-term health improvements, yet PAD is often undetected.^11^ Screening for PAD using ankle-brachial index (ABI) measurement has been advocated, but the benefits for asymptomatic individuals are not established; tests may be falsely negative without additional simple exercise tests.^12–15^ In contrast to other forms of cardiovascular disease, a large proportion of the UK PAD population do not currently receive guideline-recommended secondary prevention treatments such as antiplatelet drugs or statins.^2,16^ Current guidelines emphasise thorough history-taking and physical examination as key steps in PAD diagnosis, and the National Institute for Health and Care Excellence (NICE) recommends ABI testing when PAD is suspected.^10^ However, workload and time pressures in primary care consultations challenge guideline adherence and it is unknown as to what extent PAD guidelines are followed.^17^

The condition is poorly understood by patients and the public in comparison with cardiovascular disease. In our recent large study surveying more than 9000 members of the public, approximately two-thirds of responders reported no familiarity with PAD.^18^ The opportunity to address symptoms is most meaningful to patients, whereas clinicians recognise PAD as a vascular disease, and tend to focus on interventions to modify future cardiovascular risk.^5,19^

A group of vascular and primary care experts from 10 European countries came together in 2019 as a research group to examine care of PAD across Europe (EuroPAD). We set out to survey patient understanding of PAD, and to assess GPs’ attitudes to diagnosis and follow-up of people with PAD.^18^ A GP survey was designed, piloted, and translated for distribution across Europe. This article reports findings from the English language version distributed across England and the Republic of Ireland (RoI) during 2020.

## Method

### Survey

The survey was designed iteratively by the EuroPAD writing group, with reference to current PAD guidance in 2019, reaching consensus on questions that could reasonably assess primary care practice across Europe.^9^ Following piloting by, and feedback from, local GP colleagues, the survey was translated for use in the group’s member countries. The survey sought descriptive information about the responders’ practices and evaluated their approaches to assessment and management of PAD through a series of likelihood ratings (from 0, never, to 10, always) and discrete choice questions (Supplementary material).

The English language survey was established on the Snap Surveys online platform. A link to the online questionnaire was distributed electronically with a letter of invitation. Multiple distribution methods were used. Clinical Research Networks distributed invitations to practices across England. We also included invitations to participate in email newsletters of the Royal College of General Practitioners (RCGP) Tamar Faculty, and the RCGP Rural Forum. In the RoI, invitations were distributed by the RCGP (RoI Faculty) and the University of Limerick Research and Education Network for General Practice.^20^ Repeated social media reminders were used to raise awareness of the survey.

### Recruitment

The survey was open to all qualified GPs or GP specialty trainees in the final year of training. Invitations began in January 2020, but recruitment was paused by the sponsor as part of the COVID-19 pandemic research prioritisation process 2 months later. Recruitment was resumed in August 2020 and the survey closed in January 2021.

### Analysis

Survey responses were exported to an Excel spreadsheet. Data were analysed using Stata SE (version 16.0). Findings were summarised as proportions or median and interquartile ranges (IQR).

## Results

In total, 112 responses were received; one was uncompleted, so 111 were analysed. Sixty-eight (61%) from England and 43 (39%) from the RoI. Median (IQR) time taken to complete the survey was 6.6 (4.8–8.3) minutes. The median age of responders in the RoI was 46 (40–57) years compared with their English counterparts (median age 42 [38–50] years). Eighty-four per cent of practices in the RoI had five or less GPs, while in England this was 22%, and they were more often rural or semi-rural (Table 1). Virtually all practices (99%) had practice nurses in their teams. However, while approximately half of English practices had nurse prescribers, nurse practitioners, and/or practice pharmacists in their teams, these allied health professionals were only present in ≤7% of RoI practices.

**Table 1.** Characteristics of participating practices

	Total	England	Republic of Ireland
Completed surveys: *n* (%)	111	68 (61.3%)	43 (38.7%)
Median (IQR) age of responders		42 (38–50)	46 (40–57)
*Rurality; n = 109 (%)*		*n* = 66	*n* = 43
Rural	22 (20.2%)	12 (18.2%)	10 (23.3%)
Semi-rural	44 (40.4%)	22 (33.3%)	22 (51.2%)
Urban	43 (39.5%)	32 (48.5%)	11 (25.6%)
			
*Other GPs in practice; n* = 108 *(%*)		*n* = 65	*n* = 43
None (single-handed)	3 (2.8%)	0	3 (7.0%)
1–5	47 (43.5%)	14 (21.5%)	33 (76.7%)
6–9	40 (37.0%)	34 (52.3%)	6 (14.0%)
≥10	18 (16.7%)	17 (26.2%)	1 (2.3%)
			
*Allied health professionals in practice; n* = 109 *(%*)		*n* = 66	*n* = 43
Practice nurses	108 (99.1%)	65 (98.5%)	43 (100%)
Nurse prescribers	38 (34.9%)	35 (53.1%)	3 (7.0%)
Nurse practitioners	32 (29.4%)	31 (47.0%)	1 (2.3%)
Practice pharmacists	36 (33.0%)	36 (54.6%)	0 (0%)

The proportion of patients aged >60 years with diagnoses of hypertension, diabetes, chronic kidney disease, or coronary artery disease was similar; there was a trend towards higher prevalence of PAD in the RoI compared with England (Supplementary Table S1).

For practices in England and the ROI, when asked to consider the example of a 65-year-old man who formerly smoked, with no relevant clinical history attending for review, the median GP rating of the likelihood of enquiring about shortness of breath or chest pains was higher, at 7/10, than for enquiry about palpitations (5/10), calf and leg pains (3/10) or erectile dysfunction (2/10; Supplementary Table S2). On examining the same man, GPs would always measure blood pressure in one arm (10/10) but rarely considered checking both arms (2/10; Supplementary Table S3).

**Figure 1.**
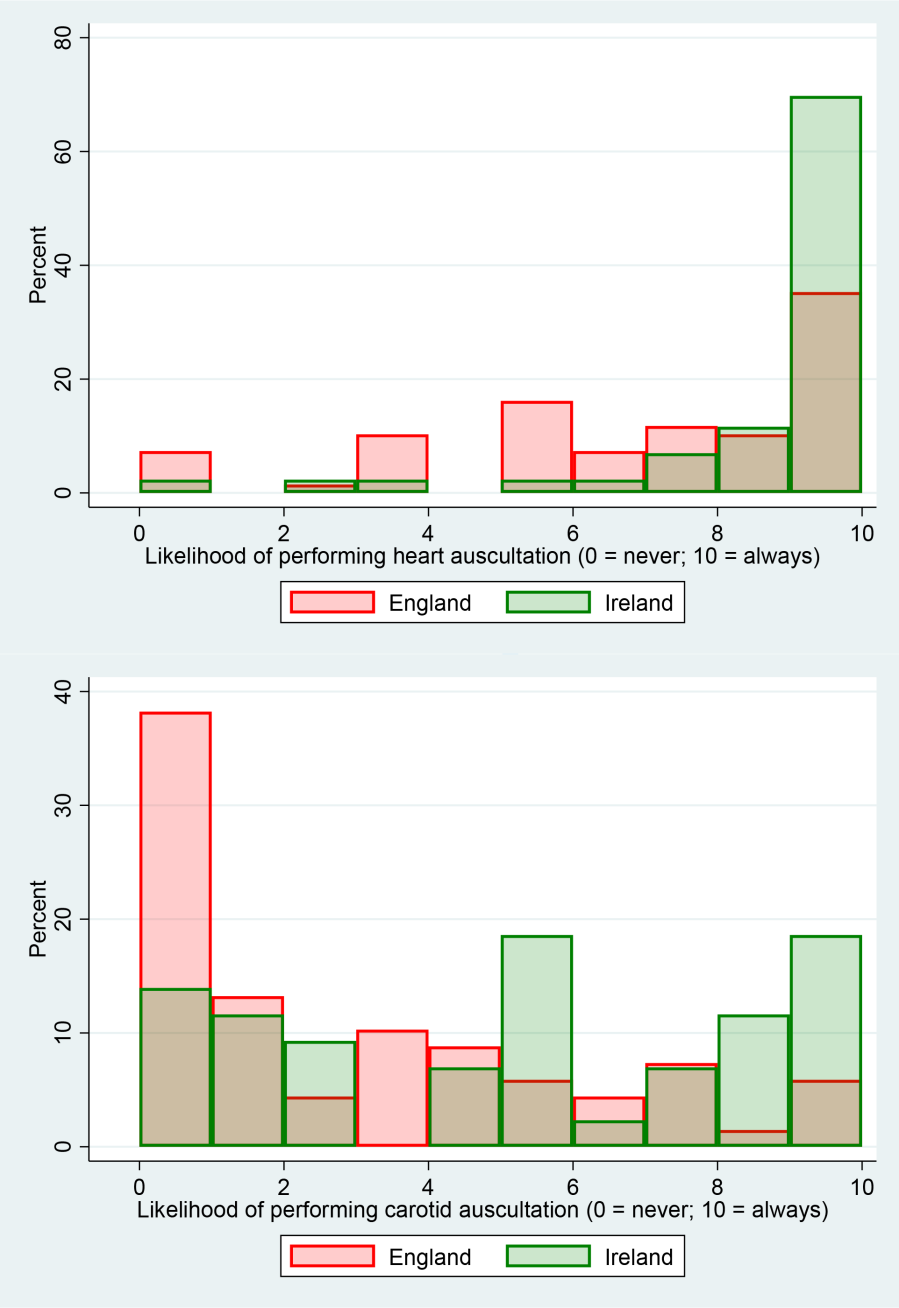
Likelihood of (a) heart and (b) carotid auscultation by English and Irish GPs on reviewing a 65-year-old man for vascular risk

GPs were more likely in the RoI than in England to perform cardiac (10/10 versus 7/10) or carotid (5/10 versus 1/10) auscultation (Figure 1, Supplementary Table S3). GPs would rarely consider palpating lower extremity pulses in this scenario (2/10; Supplementary Table S3). In general, palpation of lower limb pulses was only considered when faced with symptoms or signs of PAD, rather than as a part of any routine or systematic assessment (Table 2).

**Table 2.** Reasons for palpating lower limb pulses

*When do you palpate pulses in the lower limbs of an adult patient?*	England	RoI	Total
I almost never perform pulse palpation. If I suspect lower extremity PAD, I refer directly to a specialist	0	1 (2.3%)	1 (0.9%)
Only with claudication or rest pain in patients with CV risk factors	17 (25.0%)	6 (14.0%)	23 (20.7%)
Only in the presence of symptoms suggesting lower extremity PAD	50 (73.5%)	36 (83.7%)	86 (77.5%)
Systematically in all patients aged >65 years	0	0	0
Systematically in all my patients at least once a year	1 (1.5%)	0	1 (0.9%)
Total	68	43	111

CV = cardiovascular. PAD = peripheral arterial disease. RoI = Republic of Ireland

**Figure 2.**
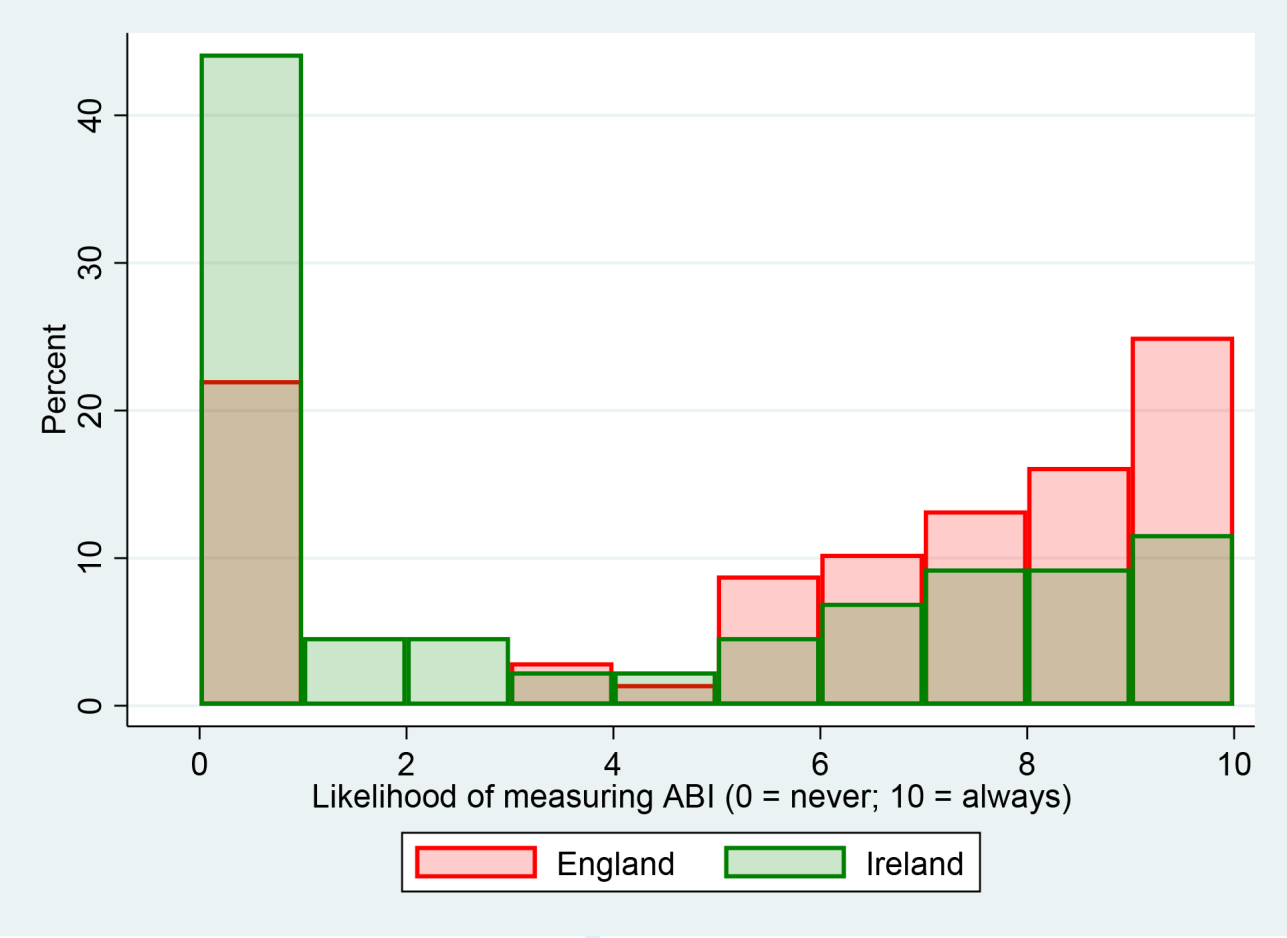
Likelihood of measuring ankle brachial index when leg pain symptoms are reported

Twenty five per cent of practices in England and 55% in the RoI reported that they do not measure ABI. Those that do measure it, predominantly use Doppler devices rather than oscillometric ones (Table 3). The single consistent scenario within which ABI measurement was reported as being undertaken was when symptoms of leg pain were present (median ratings 7/10 in England versus 2/10 in the RoI; Figure 2, Table 4). Any other indication for assessing ABI in at-risk asymptomatic individuals was rated as very unlikely (median 0/10; Table 4).

**Table 3.** Measurement approaches for ankle-brachial index

*How do you, or your practice, measure the ABI?*	England	RoI	Total
I don’t perform ABI in my practice	17 (25.0%)	23 (54.8%)	40 (36.4%)
Other	7 (10.3%)	1 (2.4%)	8 (7.3%)
Using a Doppler device and separate blood pressure cuff sequentially	39 (57.4%)	17 (40.5%)	56 (50.9%)
Using a two-limb oscillometric device (electronic sphygmomanometer) simultaneously	4 (5.9%)	1 (2.4%)	5 (4.6%)
Using an oscillometric device (that is, an electronic sphygmomanometer) sequentially	1 (1.5%)	0	1 (0.9%)
Total	68	42	110

ABI = ankle-brachial index. RoI = Republic of Ireland

**Table 4.** Likelihood of using ankle-brachial index

*In your surgery, how often do you use the ankle-brachial index in these situations:*				
	When a patient complains of pain in the legs	In asymptomatic patients who have diabetes mellitus	In asymptomatic patients with other cardiovascular risk factors	In asymptomatic patients known to have other atherosclerotic disease
Combined	6 (0–8)	0 (0–2)	0 (0–1)	0 (0–1)
England	7 (3.5–8.5)	0 (0–2)	0 (0–2)	0 (0–2)
Republic of Ireland	2 (0–7)	0 (0–2)	0 (0–0.5)	0 (0–0)

When PAD was suspected owing to intermittent claudication or leg ulcers, GPs generally (72%) referred patients to a vascular specialist, rather than requesting further investigations, to establish a diagnosis (Supplementary Tables S4 and S5). The majority (62%) also referred, rather than investigated, when PAD was suspected in the absence of symptoms (Supplementary Table S6). Referral was essentially always to a vascular surgeon as opposed to any other specialty (Supplementary Tables S7 and S8). In terms of follow-up, most GPs (86%) would regularly follow-up patients themselves after any endovascular or open revascularisation, while leaving vascular surgeons to manage their own follow-up in secondary care (30%) or re-referring as required (45%).

## Discussion

### Summary

This study describes current practice among GP responders in England and the RoI when investigating and diagnosing suspected PAD. The spectrum of disease and comorbidity is similar across the two populations served, although some professional differences in practices between the two countries emerged. Suspicion of, and investigation for, PAD seems almost entirely driven by recognition of ‘classical’ leg claudication as a trigger, whereas vascular risk factors for PAD alone generally seem to elicit little consideration. ABI measurement as a diagnostic tool is not offered in 25% of English and 55% of RoI practices, suggesting that a proportion of vascular referrals must be made based on history and physical examination findings alone, unsupported by any formal investigation.

### Strengths and limitations

This study sampled a population of GPs across England and the RoI. The distribution of practice demographics reported is consistent with existing data.^20^ As such, we consider that the results are likely to reflect the everyday practice of these GPs in suspecting, diagnosing, and managing PAD across these two countries.

Survey distribution was suspended shortly after launching in 2020 by the study sponsor, in response to the emerging COVID-19 pandemic. Other European response rates to the survey varied between countries (unpublished data) and was differentially impacted by the pandemic, limiting conclusions that could be drawn internationally. Highest numbers of responses were obtained from England and the RoI and form the basis of this article, thus summarising results for the English language version of the survey. From our previous survey experience, we anticipated obtaining 300 responses from practices across the two countries.^21^ However, reprioritisation of workloads during the COVID-19 pandemic had a substantial impact on the numbers of responses obtained. These were sufficient to support the descriptive analyses presented, but precluded planned linkage to Quality Outcome Framework (QOF) outcome data for secondary analyses.

### Comparison with existing literature

A recent systematic review exploring knowledge about PAD included no studies with GP participants from the UK, confirming a gap in this area of the literature.^22^ Suboptimal primary care measurement of ABI, as reported here, is also seen in other countries: 58% of GPs in Australia previously reported never measuring ABI; a Dutch study found poor adherence of physicians to their own PAD guidelines with suboptimal ABI assessment; and in France, only 42% of GPs surveyed knew that ABI was recommended by their health authorities for detection of PAD.^23–25^ While intervention for PAD is incentivised by the QOF in England, the fact that it is not included in the Chronic Disease Management Programme in the RoI might influence the differences in ABI uptake reported between the two countries here.^26,27^

Awareness of PAD is also poor in the general population; our recent international survey of more than 9000 members of the public found that the majority (57%) reported no familiarity with PAD, and 55% were unaware of the potential limb consequences of PAD.^18^ Leg symptoms vary and are not always those classically recognised as intermittent claudication.^2,7^ Even textbook symptoms of calf pain on exertion are often attributed by patients to ageing or other processes, leading to delays in the presentation and diagnosis of PAD.^28^

### Implications for research and practice

Differing auscultation rates between GPs in England and the RoI may reflect different cultural expectations of patients between the two countries, as well as differential access to diagnostic tests. The variability in presentation and understanding of the symptoms of PAD are likely to be contributing factors to the under-reporting and underdiagnosis of PAD.^8,18^ Prevalence of PAD is higher in those from lower socioeconomic groups, particularly in men.^29^ These groups tend to also have higher cardiovascular risks and engage less frequently with cardiovascular screening programmes.^30^ Therefore, doctor-led enquiry after symptoms beyond leg pain is required to better identify potential cases of PAD.

Targeted screening for PAD in primary care using ABI measurement has previously been advocated and has been shown to be feasible; however, in practice, it does not take place.^31^ When PAD is suspected, ABI measurement is recommended in NICE and European guidelines to confirm the diagnosis.^9,10^ The current findings suggest that this is not always implemented in England or the RoI. Existing time constraints and lack of staff availability have been exacerbated by rising workload, making it difficult to adopt procedures, such as ABI, formerly regarded as secondary care investigations.^32^ Lack of training is another reason for limited use of ABI for PAD diagnosis; targeted training of nursing staff has been advocated to improve ABI measurement uptake,^23,33^ which could be extended to other allied healthcare professionals. However, as reflected in our findings, in the RoI integration of community pharmacists into practices is, at present, novel, and there is no recognised training scheme for practice nurses or advanced practitioners. The reported low likelihood of bilateral brachial blood pressure measurement being undertaken suggests incomplete adherence to recommended approaches to ABI measurement, and represents further missed opportunities to recognise cardiovascular risk markers and thus optimise secondary cardiovascular prevention.^2,34–37^

In conclusion, this survey has highlighted missed opportunities to identify PAD in primary care in England and the RoI, owing to under-recognition of the full extent of cardiovascular risk markers and the range of symptoms associated with PAD. Universal access to confirmatory ABI testing to diagnose PAD is lacking in primary care, suggesting that cases may be missed or inappropriately referred to secondary care. Although the sample of participants was self-selected, we believe the findings support a preliminary call for a programme to raise awareness about symptoms and diagnosis of PAD aimed at improving care for this relatively under-recognised and undertreated group.
